# Systematic evaluation of commercially available pain‐management mHealth apps for chronic pain in the United Kingdom

**DOI:** 10.1111/bjhp.70053

**Published:** 2026-01-26

**Authors:** Rebecca P. Harding, Jenna L. Gillett, Michael Passaportis, Eleanor Miles, Faith Matcham

**Affiliations:** ^1^ School of Psychology University of Sussex Falmer UK; ^2^ Department of Psychology University of Warwick Coventry UK; ^3^ School of Psychology University of Buckingham Buckingham UK

**Keywords:** adaptivity, behaviour change techniques, mHealth, pain apps, self‐management

## Abstract

**Objectives:**

Self‐management is central in chronic pain care, and mobile health (mHealth) applications (apps) offer scalable tools to support symptom monitoring and management. Although promising, these apps vary in quality, adaptability, and integration of evidence‐based behaviour change techniques (BCTs). Many remain unregulated and under‐evaluated, leaving their benefits for pain management unclear. We systematically evaluated the quality of commercially available pain management apps in the United Kingdom and examined the prevalence of pain‐related BCTs and adaptive features.

**Design and Methods:**

Freely available English‐language apps from the ‘Health and Fitness’ or ‘Medical’ categories in the Apple® and Google Play® stores were screened and assessed for quality using the Mobile App Rating Scale (MARS; 1 = inadequate, 5 = excellent) and coded for BCTs and adaptive features.

**Results:**

Twenty‐three apps were included, with a mean MARS score of 3.03 (range = 1.8–4.6). Five scored >4.0, while 39% scored 3.0–3.9, indicating moderate quality. Apps included a mean of 3.3 BCTs, most commonly self‐monitoring (87%), instruction (61%), and behaviour–health links (52%). Social support (13%) and goal setting (17%) were rare. An average of 2.3 adaptive mechanisms were identified, with proximal outcomes in all apps and intervention options in 70%, but decision points and tailoring variables were infrequent.

**Conclusion:**

Commercially available pain apps in the United Kingdom are generally of moderate quality, with limited integration of social, goal‐setting, and adaptive features. Greater personalization is needed to strengthen engagement and clinical impact in digital pain self‐management.

**Statement of Contribution**

**What is already known on this subject?**

- mHealth tools support scalable self‐management in chronic pain care.
- Pain apps remain unregulated and under‐evaluated, leaving their efficacy unclear.
- Digital interventions' quality, behaviour change, and adaptivity shape engagement.

**What does this study add?**

- Most freely available UK pain apps are of moderate quality.
- Pain apps rarely use key Behaviour Change Technique like social support or goal setting.
- Increased adaptability and personalization are critical for engagement.

## INTRODUCTION

Chronic Pain is a complex condition sustained by interacting biological, psychological and social mechanisms rather than purely biomedical processes (Topcu, 2018). Psychological and social factors do not simply arise as consequences of pain—they actively shape pain perception, coping, and long‐term outcomes across all pain types. Because behavioural and psychosocial processes are central to chronic pain trajectories, contemporary guidelines advocate a biopsychosocial approach that prioritizes multi‐disciplinary and behaviour‐focused interventions (Angst et al., 2020; National Institute of Care and Excellence, 2021). NICE advises against utilizing pharmaceutical intervention alone for chronic pain, due to the high risk of unwanted side‐effects, opioid addiction and disregard for the inter‐relation of psychological/social factors in pain experiences (Cheatle, 2016; Darnall et al., 2018; Miaskowski et al., 2020). However, achieving optimal multi‐faceted treatment remains challenging within the constraints of individual health care systems (British Medical Association, 2025). Self‐management strategies fall under the recommended multi‐disciplinary approach for pain management and are considered an effective non‐pharmacological approach (National Institute of Care and Excellence, 2021). Typically, self‐management strategies involve activities such as low‐impact exercise, meditation, mindfulness and pain tracking that patients can practise independently, outside of clinical settings (Svendsen et al., 2020).

Mobile health (mHealth) interventions represent an effective way to deliver self‐management support (Fisher et al., 2019; Gamwell et al., 2021). mHealth has the potential to enhance accessibility and address treatment delays, with functions including education, self‐monitoring and treatment (Devan et al., 2019). Research findings have suggested that these tools empower patients, improve understanding of their condition, and enhance pain‐related outcomes (Najm et al., 2019). However, systematic review evidence has highlighted that despite their potential efficacy, mHealth used by patients can incur attrition rates of up to 50% for internet‐based chronic pain interventions (Buhrman et al., 2016), and more recent research posits similar disengagement with app‐delivered interventions (Eaton et al., 2024). One explanation for such high attrition and disengagement is that digital health tools are not consistently designed in line with best practises for behaviour change, limiting their sustained impact (Cucciniello et al., 2021). Furthermore, research in this area often demonstrates limited diversity and consistency in how ‘engagement’ is defined and measured for digital health interventions, with many studies omitting engagement in metrics altogether (MacLean et al., 2025).

Engagement with pain self‐management is a complex health behaviour involving a dynamic set of emotional, behavioural and emotional processes (Antunes et al., 2024; Vase et al., 2025). Previous work has highlighted shortcomings in app design—including limited use of evidence‐based behaviour change techniques (BCTs), oversimplified functionality, and insufficient testing (Lalloo et al., 2015, 2017; Silva et al., 2020)—the current evidence base remains fragmented. Evaluations often focus on narrow app samples, single pain conditions or non‐UK context, limiting their generalizability and leaving health care professionals with little consolidated guidance on which tools are recommended (De La Vega & Miró, 2014; Gamwell et al., 2021). Although some elements valued by patients, such as goal setting and social support, are grounded in BCT theory (Michie et al., 2011), their implementation across applications (apps) is inconsistent and underexplored (Gamwell et al., 2021; Harding et al., 2026; Magee et al., 2021). While personalization and adjunctive use appear to enhance patient experiences when deployed alongside in‐person care (Main et al., 2025), the extent to which these features are embedded within UK‐published pain apps is unclear. Recent reviews show mixed findings: US‐based pain apps demonstrated variable quality and inclusion of pain‐specific BCTs (Gamwell et al., 2021), whereas a UK review restricted to low back pain only reported poor quality and limited theoretical underpinnings (Zhou et al., 2024). This work highlights the absence of a comprehensive synthesis of UK‐published pain management apps across chronic pain conditions—a gap this systematic review seeks to address.

Beyond quality and the inclusion of BCT principles, a further underexplored dimension of pain self‐management tools regards their capacity to adapt to users' fluctuating needs. For people living with chronic pain, symptoms, motivation and self‐management requirements can change considerably across the day, meaning static or one‐size‐fits‐all approaches are insufficient (Wesolowicz et al., 2021). MHealth tools theoretically offer the ability to tailor support dynamically in response to individual progress, context, or reported experiences, yet it remains unclear to what extent current pain‐management apps integrate such adaptability into their design. In other areas of health, adaptive features have been associated with greater engagement and improved outcomes (Collins et al., 2024; Hardeman et al., 2019; Perski et al., 2022); similar functionality could enhance the effectiveness of pain apps. Evaluating whether existing tools harness this adaptive potential alongside evidence‐based BCTs is therefore warranted.

Based on the inconsistent evidence regarding the quality and theoretical grounding of pain‐management apps, alongside the underexplored potential of adaptability to meet patients' fluctuating needs, this review addresses two questions:

1. What is the quality of UK mHealth apps for chronic pain self‐management, and which pain‐relevant BCTs do they employ?
2. To what extent do these apps incorporate adaptive features to support self‐management?

## METHODS

### Protocol and registration

This study implemented a systematic review of apps published in Apple Store and Google Play, with a protocol and data extraction procedure designed to align with the Preferred Reporting Items for Systematic reviews and Meta‐Analyses (PRISMA) (Moher et al., 2009; Page et al., 2021). Given the focus on commercially available apps rather than clinical trial evidence, several checklist items (e.g., risk of bias, quantitative synthesis) were not applicable and are indicated as such in the completed PRISMA checklist. The protocol was prospectively registered on the Open Science Framework (OSF) in February 2024 (Harding et al., 2024). Minor deviations from the preregistered protocol were made during the review process, outlined and justified in Data S1. Identification of eligible apps, as well as full screening procedures, were conducted independently by two authors (RPH and JLG) before inter‐rater reliability checks. All data were stored using an Excel spreadsheet accessible to the raters, and detailed procedure logs were maintained. Apps identified for review were coded independently by the raters. Following initial coding, a mediation session was conducted, during which the raters discussed their scores, engaged in reflective conversations to challenge any disagreements in ratings, and reached consensus for each app and scoring variable. Where necessary, a senior author (FM) was available to resolve disputes and act as a tiebreaker.

### Eligibility criteria

Apps were included if they met the following criteria: (i) free to download, (ii) available in English, (iii) listed under ‘Health & Fitness’ or ‘Medical’ categories on either store, (iv) specifically referenced ‘pain’ in their app description, and (v) incorporated a form of self‐management. Apps were excluded if they were (i) designed for HCPs rather than patients or (ii) if they required specialist devices or equipment for use (e.g. TENS machine). Screeners read the descriptions of each app provided on the app stores and, based on the information given for each app, a decision was made by each rater independently as to whether the app was suitable for inclusion or exclusion.

The decision to include only freely available apps was informed by previous evaluations (Gamwell et al., 2021) and preliminary qualitative interviews, in which individuals with chronic pain described financial barriers, particularly subscription costs, as a major deterrent to engagement. This reflects broader evidence of socioeconomic disadvantage in this population and ensured the review focused on accessible, relevant tools (De Sola et al., 2016; Wu et al., 2017).

### Systematic search

A series of five systematic searches were conducted between the dates of February and July 2024. The terms ‘chronic pain’, ‘pain management’, ‘chronic pain patients’ and ‘reduce pain’ were entered into the search bar in the Apple IOS App Store and Google Play. The abbreviation ‘CPPs’ was also tested for completeness, although this term is rarely used in consumer‐facing app descriptions and did not yield additional results. We did not include condition‐specific terms (e.g., fibromyalgia, low back pain, arthritis, headache, cancer‐related pain) as the review aimed to capture apps marketed for general chronic pain self‐management rather than individual conditions. As each of the two reviewers conducted these searches independently in both stores, this amounted to a total of 20 systematic searches. A list of the first appearing 25 apps for each of these search terms was recorded as Step 1 of this review, aligning with consumer research findings <15% of consumers download an app that did not appear in the top 10 or at least in the top 25 (Chavez et al., 2017; Ramsey et al., 2019). Additionally, the approach supports the algorithms of both app stores where preference for download is shown to the top 25 apps listed.

A re‐screening phase took place between December 2024 and January 2025 to verify the continued eligibility of apps (Step 2). This step was essential due to the dynamic nature of app stores, with apps updated, removed, rebranded or changed over time. Once all searches were complete, duplicates were removed (Step 3) before applying inclusion/exclusion criteria (Step 4). Separate logs for search results were kept, ensuring transparency between raters to ensure independent ratings in the first instance. Once the final list of apps that met inclusion/exclusion criteria was determined for each store, each rater moved on to the next phase of review, downloading and piloting individual apps (Step 5), allowing for detailed assessment and scoring for the key outcomes.

### Data extraction and quality assessment

The key data analyzed for this review were sourced from (i) information about the apps on page descriptions and (ii) first‐hand piloting of apps for functionality and exploration of content. The following key variables were explored: quality measure of the app across multiple domains, presence and type of BCTs identified within the app, and adaptive features identified within the app.

#### Quality assessment (Mobile App Rating Scale [MARS]) (Stoyanov et al., 2015)

App quality was assessed using the validated 23‐item MARS (Stoyanov et al., 2015), a widely used instrument for evaluating the quality of MHealth apps. This replicates Zhou et al. (2024) who applied the MARS as a measure of quality of pain management apps for lower back pain. MARS includes four core domains: Engagement; Functionality; Aesthetics; and Information Quality, each rated on a five‐point Likert scale (1 = inadequate quality, 5 = excellent quality; Stoyanov et al., 2015). Domain scores were calculated by averaging the items within each domain, and an overall MARS quality score was obtained by averaging the four‐domain means. For interpretability of overall mean scores, we categorized means as follows: 1.0–1.9 = inadequate; 2.0–2.9 = poor; 3.0–3.9 = acceptable; 4.0–4.4 = good; and 4.5–5.0 = excellent. These revised categories acknowledge the rarity of apps achieving a full score of 5 across domains. Two optional subscales, Subjective Quality and App‐Specific Quality, were also included to assess rater satisfaction and the perceived impact of the app on health‐related behaviour change. These were scored using the same scale and reported separately, in accordance with MARS scoring guidelines. While MARs (Stoyanov et al., 2015) captures usability and design quality, it does not assess theoretical content or adaptability. An app may score highly on MARS yet lack evidence‐based BCTs or adaptive features or conversely include multiple BCTs but perform poorly on engagement or functionality. Evaluating these components separately provides a fuller picture of app strengths and limitations.

#### Identification and coding of BCTs

To identify BCTs relevant to pain self‐management, we followed the approach outlined by Gamwell et al. (2021). Their review identified eight core BCTs consistently associated with pain management efficacy: behaviour‐health link; consequences; instruction; prompt intention formation; prompt‐specific goal setting; self‐monitoring; social support/change; stress management. These BCTs formed the basis of our coding framework and were used to assess the presence or absence of pain‐specific BCTs within each app. As Gamwell et al.'s review represents the only published synthesis of pain‐specific BCTs to date, their findings were used as a standard for determining relevant and evidence‐based techniques.

#### Identification and coding of adaptive features

To identify presence (or absence) of adaptive features and personalization options within each app, an adaptive features checklist was created by the research team, based on previous research pertaining to the requirement of adaptive interventions (Nahum‐Shani et al., 2015, 2016). The presence or absence of these adaptive features was recorded: distal outcomes (long‐term goals of the intervention, such as improved pain management), proximal outcomes (short‐term indicators of progress), decision points (moments when the app can deliver support), intervention options (different types of support or content the app can provide), tailoring variables (user characteristics or data used to personalize delivery), and decision rules (the guidelines that link tailoring variables to intervention options). Coding was guided by established definitions and frameworks for adaptive features to evaluate how apps might adaptively support users in real‐time contexts (Collins et al., 2024; Nahum‐Shani et al., 2015, 2016).

### Scoring and analyses

An overall MARS quality score was calculated as the mean of the four objective subscales (Engagement, Functionality, Aesthetics, and Information; Stoyanov et al., 2015). The two optional subscales—Subjective Quality and App‐Specific Quality were also rated but were treated as exploratory outcomes and reported separately. In addition, a composite score was created to capture a broader picture of app utility, integrating (i) the overall MARS quality score (objective subscales only), (ii) the total number of pain‐related BCTs coded per app, and (iii) the total number of adaptive mechanisms identified per app. The resulting composite score provided an integrated indicator of app quality, theoretical underpinning, and adaptability, offering insight into the potential effectiveness of each pain‐management app.

### Inter‐rater reliability and competence of raters

Two independent raters (RPH and JLG) conducted searches and assessments of apps. Each rater has had training and experience in behaviour change theory and intervention development at postgraduate level and was well‐equipped to identify relevant components for this review. The process was supervised by a Chartered Health Psychologist with expertise in digital intervention design (FM) who was available to resolve disagreements. Each coder engaged with each app for a minimum of 10 min before independently recording MARS scores, BCTs, adaptive‐related codes, and other app features, consistent with recommendations for mHealth app evaluation (Stoyanov et al., 2015). During this period, coders reviewed the full app‐store description and systematically explored all accessible features by completing onboarding or educational material and interacting with key functions such as symptom‐tracking tools, exercises or self‐management modules. Coding was documented in structured extraction forms, with BCTs and adaptive elements coded for presence or absence based solely on directly observable content and functionality.

This engagement and coding workflow aligns with procedures used in prior app evaluations (Gamwell et al., 2021; Ramsey et al., 2019), in which reviewers independently interacted with apps for a minimum of 10 min, coded observable features, and resolved discrepancies through discussion.

## RESULTS

**FIGURE 1:**
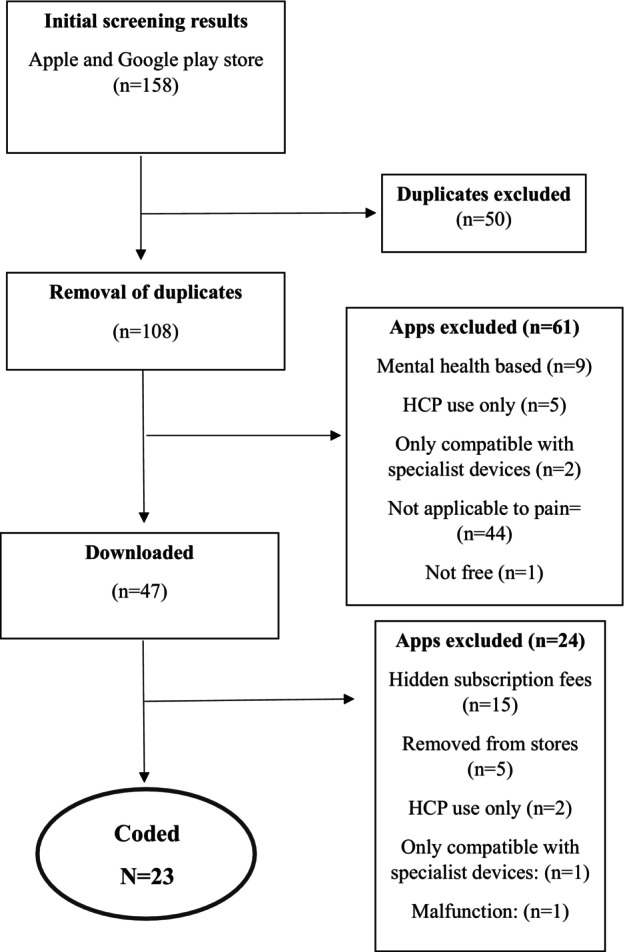
PRISMA flow diagram.

Our search across Apple Store and Google Play initially identified 158 apps, of which 23 were coded for quality, pain relevant BCTs and adaptive features (Figure 1).

### App quality (MARS ratings)

Analysis of the 23 pain‐management apps revealed considerable variability in overall quality as rated by the MARS (Table 1). Overall MARS scores ranged from 1.8 to 4.6, with a mean of 3.03, indicating that most apps on average fell within the moderate quality range. Only five apps (22%) achieved high‐quality scores above 4.0: Pathways Pain Relief and Bodyguide Pain Relief Exercises (both 4.6), Wave Health: Symptom Tracker (4.1), Arthritis Tracker (4.0) and Manage My Pain (4.0). Nine additional apps (39%) scored between 3.0 and 3.9, indicating moderate quality, while the remaining apps fell between 2.0 and 2.9 (26%), indicating poor quality, and 1 and 1.9 (13%) indicating inadequate quality.

**TABLE 1:** App demographic characteristics and MARs quality indicators.

App name	Platform^a^	Purpose	N. Reviews	Developer	Engagement score	Functionality score	Aesthetics score	Information score	Mean MARS	MARS optional domains	
										Subjective	Specific
Pathways Pain Relief	A/G	Pain Therapy programme	28	Pathways	4.8	5.0	4.6	4.0	4.6	4.0	3.5
Bodyguide Pain relief exercise	A/G	Physical exercises and massage	0	Body Guide	4.8	4.5	4.7	4.2	4.6	4.3	4.8
Wave Health: Symptom tracker	A/G	Symptom tracker	13	Treatment technologies and insights LLC	4.2	4.5	4.7	2.9	4.1	2.8	2.2
Manage my pain	A/G	Tracking pain and SMS	194	Managing Life	4.4	4.5	4.0	3.6	4.0	4.5	4.0
Arthritis Tracker	A/G	Tracking and psychoeducation	16	Versus Arthritis	3.6	5.0	4.3	3.1	4.0	3.3	3.8
Tracker, Reminder‐CareClinic	A	Health tracking and medication reminders	373	CareClinic software inc	4.0	4.0	4.7	2.9	3.9	3.3	3.8
Chronic Insights symptom diary	A/G	Symptom tracking and breathing exercises	24	Chronic Insights LTD	3.6	4.0	3.7	2.9	3.6	3.3	2.3
Back Pain Relief exercises	A/G	Physical activity	1	Samantha Roobol	2.8	4.0	4.3	3.0	3.5	1.7	2.2
Lower Back Pain Exercises	A/G	Physical exercises	2	Stefan Roobal	3.0	3.75	4.0	2.3	3.3	2.5	1.8
Exercises to reduce pain	G	Physical exercises	0	Fitric	2.8	4.75	4.3	1.4	3.3	2.8	2.2
Apo Health companion	A	Tracking	0	Apo Tech Care	3.4	3.5	4.0	2.0	3.2	2.0	3.3
Stretch exercise‐ flexibility	G	Physical exercises	107,000	Unknown	3.6	2.5	4.0	2.4	3.1	2.5	2.2
Juva Stress and Migraine	A	Treatment tracker and relaxation techniques	15	Juva Health Inc	3.2	3.25	4.0	1.4	3.0	1.8	1.3
mySymptoms Food Diary	A/G	Food diary and tracking	760	SkyGazer Labs LTD	2.4	4.75	3.0	2.0	3.0	2.0	1.8
Knee Exercises	A/G	Physical exercises	2	Stefan Roobol	2.6	4.0	3.0	2.0	2.9	2.3	2.7
Journal my Health	A/G	Tracking	1	Chariot solutions	2.9	4.25	2.0	2.0	2.9	1.5	2.0
Purity Gout diet management	A	Tracking	31	Ariana Pineda	2.8	3.5	4.0	1.0	2.8	3.3	2.7
Symptom Tracker	A	Symptom tracking	129	Adam Cziko	2.6	3.25	3.0	1.7	2.6	2.3	1.8
Ada Check your Health	A/G	Tracking and Health information	5600	Ada Health	2.6	2.75	3.7	0.7	2.4	1.5	3.8
#trackit: Track Health and Pain	A	Health and pain tracker	2	Jordi Kitto	1.4	3.75	2.0	1.1	2.0	1.3	1.8
Alogea	A	Pain Journal and medication tracking	4	Solent Anaesthesia and Pain relief services	2.4	2.0	1.7	1.6	1.9	1.3	1.5
Life notes‐ Symptom Tracking	G	Symptom tracking	544	Seb Ferrer	1.8	4.0	1.3	0.4	1.8	1.3	1.3
Painalog		Body map scanning	4	Didis Dosa LLC	2.0	1.5	1.7	0.6	1.45	1.8	1.2

*Note*: Abbreviations and variable information: MARS = Mobile App Rating Scale (Stoyanov et al., 2015); Developer name = as presented on corresponding application stores; Engagement, Functionality, Aesthetics and Information are subdomains of the MARS global quality score; App subjective score = coders' overall satisfaction and likelihood of recommending/using the app (four items: recommendation, usage, willingness to pay, overall star rating); App‐Specific Score = perceived potential for behaviour change (four items: awareness, knowledge, attitudes, and intention to change/help‐seeking). Apps are presented highest‐lowest according to overall (mean) quality (MARS) rating.

^a^ A, Apple Play Store; G, Google Play Score; A/G, Apple and Google Play.

Subscale scores followed a similar pattern. Functionality was the strongest‐performing domain across apps, with several achieving scores above 4.5 (e.g., mySymptoms Food Diary [4.75], Exercises to Reduce Pain [4.75], and Arthritis Tracker [5.0]). Aesthetics and engagement scores were more varied, with apps such as Bodyguide and Pathways performing consistently well across all domains. In contrast, information quality was more variable and often underdeveloped, particularly in apps like Painalog (0.6) and Ada Check Your Health (0.7), where educational content or clinical credibility appeared limited.

Optional MARS subscales provided additional insight into perceived value and behavioural relevance from raters. Subjective quality scores, which capture user satisfaction and likelihood of future engagement, were low across most apps (mean = 2.47). Only three apps received strong subjective ratings above 4.0: Manage My Pain (4.5), Bodyguide (4.25) and Pathways (4.0). Four additional apps, including Arthritis Tracker and Purity Gout Diet Management, received moderate scores between 3.0 and 3.9, while the majority (70%) fell below 3.0—suggesting limited appeal or usability from the end‐user perspective.

App‐specific quality scores, which reflect the coders' perceived potential to influence behaviour change, followed a similar trend (mean = 2.52). Just two apps—Bodyguide Pain Relief Exercises (4.8) and Manage My Pain (4.0)—achieved scores above 4.0. Most apps (70%, *n* = 16) were rated between 1.2 and 2.9, indicating that although many apps function adequately, few offer meaningful support for health behaviour change grounded in theory or evidence‐based strategies.

These findings suggest that while a small number of apps demonstrate high overall quality and behaviour change potential, most fall short on key components related to user engagement, satisfaction, and behavioural effectiveness. Notably, those with stronger MARS scores tended to also perform well on subjective and app‐specific domains.

### Inclusion of BCTs

Further, analysis of the 23 pain‐management apps revealed substantial variability in the integration of BCTs and adaptive intervention features, with only a small proportion demonstrating comprehensive implementation of both. Across all apps, the number of pain‐specific BCTs ranged from 1 to 6 (mean = 3.3), with just 17% (*n* = 4) employing five or more techniques. While over half of the apps (*n* = 12, 52%) included at least three BCTs, coverage of specific techniques was uneven. The most applied BCT was *self‐monitoring*, found in 87% of apps (*n* = 20), followed by *instruction* (61%, *n* = 14), *behaviour‐health link* (52%, *n* = 12), and *prompt intention formation* (52%, *n* = 12). Table 2 provides examples of how the most frequently coded BCTs were implemented in the evaluated apps. In contrast, *social support* and *specific goal setting* were notably underutilized, present in only 13% (*n* = 3) and 17% (*n* = 4) of apps, respectively. This suggests that most apps focused on self‐regulatory strategies over interpersonal or motivational supports (Table 3).

**TABLE 2:** Examples of most frequently used BCTs in apps.

BCT	Definition and examples used in apps
Self‐monitoring	The user is encouraged to regularly track or record a specific behaviour or symptom within the app (e.g., using a diary or logging tool) Users are prompted to rate their pain levels multiple times per day (e.g., morning, midday, evening), and to track associated factors such as alcohol intake and medication use (Pathways Pain Relief) Users can log food intake and symptoms, including the ability to document intake of specific foods, drinks and medications to identify potential triggers (My Symptoms Food Diary) The app allows for tracking of health‐specific biomarkers such as uric acid and purines, relevant to gout management (Purity Gout Diet Management) Users can monitor exercise and physical activity patterns, including workout frequency and type (Knee Exercises) The app provides comprehensive tracking features, allowing users to record pain, energy levels, medication, sleep, emotions and stress levels (Pathways Pain Relief)
Instruction	The app provides guidance on how to carry out a behaviour or perform preparatory actions necessary to support it Instructional content is delivered through structured learning pathways and videos guiding the user through exercises or behavioural strategies (Pathways Pain Relief) Users are advised to record symptoms and timestamp their onset for future review (My Symptoms Food Diary) The app guides users through exercises or relaxation techniques such as meditation or breathing through videos or animations (Tracker, Reminder Care Clinic) Biofeedback‐based guided meditations and exercises are provided to support stress and migraine management (Juva Stress and Migraine) Video tutorials demonstrate how to perform specific physical exercises, such as lower back stretches (Lower Back Pain Exercises)
Behaviour‐Health link	The app explains how certain behaviours are connected to health outcomes, providing information that helps users understand the benefits of engaging in specific actions Psychoeducational content explains the science behind pain and how behavioural strategies, such as movement and relaxation, affect pain perception (Pathways Pain Relief) Descriptive statements within the app content explain that daily movement and exercise can reduce pain and improve overall wellbeing (Exercises to Reduce Pain) The app discusses the benefits of behaviour change for long‐term pain outcomes, such as the value of regular physical activity in reducing chronic pain (Lower Back Pain Exercises) The app highlights the importance of exercise for joint and muscular health is communicated to users (Knee Exercises) The app provides infographics and written content demonstrate how behaviour (e.g., healthy eating, physical activity) positively influences long‐term health outcomes (Versus Arthritis Tracker)
Prompt intention formation	The app supports users in setting specific goals or making a clear commitment to perform a behaviour Users are encouraged to complete structured programmes that include goal setting, streak tracking and regular body/mind challenges to support behaviour consistency (Pathways Pain relief) Goal‐setting features allow users to establish specific targets for exercise or wellness routines, with reminders to support adherence (*Alogea*) Apps provide prompts to review personalized data reports, helping users reflect on patterns related to pain intensity and flare‐ups (*Manage My Pain*) Tracking reminders can be scheduled to prompt consistent engagement with symptom monitoring (*Wave Health: Symptom Tracker*) Habit formation is supported by streak counters and behaviour‐triggered notifications to encourage regular engagement (*Bodyguide Pain Relief Exercises*)

Abbreviation: BCT, behaviour change technique.

**TABLE 3:** Inclusion of BCTs.

App name	Behaviour health link	Consequences	Instructions	Prompt intention	Prompt specific	Self‐monitoring	Social support	Stress management	Total pain BCTs
Pathways pain relief	✕	✕	✕	✕	✕	✕		✕	7
Journal my Health	✕	✕		✕	✕	✕		✕	6
Bodyguide Pain relief exercise	✕	✕	✕	✕		✕	✕		6
Strech exercise—flexibility		✕	✕	✕	✕	✕			5
Tracker, Reminder‐CareClinic	✕		✕			✕		✕	4
Exercises to reduce pain	✕		✕			✕		✕	4
Knee Exercises	✕	✕	✕			✕			4
Ada Check your health	✕		✕	✕		✕			4
Arthritis Tracker	✕			✕	✕				4
Painalog	✕		✕			✕			3
Back pain relief exercises			✕	✕	✕				3
Symptom Tracker			✕	✕		✕			3
Manage my Pain	✕			✕		✕			3
Wave Health: Symptom Tracker	✕			✕		✕			3
Juva Stress and Migraine			✕			✕		✕	3
Alogea						✕		✕	3
mySymptoms Food Diary			✕			✕			2
Purity Gout diet management			✕			✕			2
Lower Back Pain Exercises	✕		✕						2
Chronic Insights symptom diary						✕		✕	2
#trackit: Track Health and Pain							✕		1
Apo Health Companion								✕	1
Life notes—Symptom tracking							✕		1
*Total*	12	6	14	12	4	20	3	5	

*Note*: All tables are presented highest‐lowest according to overall amount of BCTs present.

Abbreviation: BCT, behaviour change technique.

### Inclusion of adaptive features

A parallel analysis of adaptive intervention features found similarly limited integration (Table 4). While all apps included a proximal outcome, typically in the form of symptom tracking, only a subset incorporated additional adaptive mechanisms. Intervention options were present in 70% (*n* = 16) of apps and distal outcomes in 44% (*n* = 10), but more sophisticated elements such as decision variables (*n* = 2), tailoring variables (*n* = 5) and decision points (*n* = 0) were either rare or absent. Despite this, several apps stood out for their relative complexity. *Painalog*, for example, included five adaptive mechanisms (i.e., distal outcome, proximal outcome, tailoring variables, intervention options, decision variables), while *Bodyguide Pain Relief Exercise*, *Apo Health Companion* and *Wave Health: Symptom Tracker* each incorporated four.

**TABLE 4:** Inclusion of adaptive features.

App name	Adaptive mechanism						
	Distal outcome	Proximal outcome	Decision points	Tailoring variables	Intervention options	Decision variables	Total adaptive mechanisms
Painalog	✕^a^	✕		✕	✕	✕	5
Apo Health Companion	✕^a^	✕		✕	✕		4
Wave Health: Symptom Tracker		✕		✕	✕	✕	4
Bodyguide Pain relief exercise	✕	✕		✕	✕		4
Pathways pain relief	✕	✕			✕		3
Exercises to reduce pain	✕	✕			✕		3
Arthritis Tracker	✕	✕			✕		3
#trackit: Track Health and Pain		✕		✕^a^			2
Tracker, Reminder‐CareClinic		✕			✕		2
Alogea	✕	✕					2
Juva Stress and Migraine		✕			✕		2
Purity Gout diet management		✕			✕		2
Lower Back Pain Exercises		✕			✕		2
Knee Exercises		✕			✕		2
Manage my Pain	✕^a^	✕					2
Chronic Insights symptom diary		✕			✕		2
Journal my health	✕^a^	✕					2
Ada Check your health		✕			✕		2
Life notes—symptom tracking	✕^a^	✕					2
Strech exercise—flexibility		✕		^a^	✕		2
Back pain relief exercises		✕		^a^	✕		2
mySymptoms Food Diary		✕					1
Symptom Tracker		✕					1
Total	10	23	0	5	16	2	

*Note*: All tables are presented highest‐lowest according to overall number of adaptive mechanisms.

^a^ Implied, rather than explicitly stated.

When considered together, these behavioural and adaptive features formed the basis for a composite score, designed to reflect each app's overall potential as a digital health intervention (maximum score = 19; Table 5). The highest‐scoring apps—*Pathways Pain Relief* and *Bodyguide Pain Relief Exercise* (both 14.6), followed by *Wave Health: Symptom Tracker* (11.1) and *Arthritis Tracker* (11.0)—combined a relatively high number of BCTs with stronger adaptive capacities and favourable MARS ratings. Conversely, lower‐performing apps such as *Life Notes – Symptom Tracking* (4.8), *#trackit: Track Health and Pain* (5.0) and *mySymptoms Food Diary* (6.0) demonstrated limited use of both BCTs and adaptive mechanisms, suggesting lower potential for behaviour change support or personalized delivery.

**TABLE 5:** Composite scores (quality, BCT and adaptive feature inclusion).

App name	MARS quality	Total pain BCTs	Total adaptive mechanisms	Total scores
Pathways pain relief	4.6	7	3	14.6
Bodyguide Pain relief exercise	4.6	6	4	14.6
Wave Health: Symptom Tracker	4.1	3	4	11.1
Arthritis Tracker	4	4	3	11.0
Journal my health	2.9	6	2	10.9
Exercises to reduce pain	3.3	4	3	10.3
Strech exercise—flexibility	3.1	5	2	10.1
Tracker, Reminder‐CareClinic	3.9	4	2	9.9
Painalog	1.45	3	5	9.5
Manage my Pain	4	3	2	9.0
Knee Exercises	2.9	4	2	8.9
Back pain relief exercises	3.5	3	2	8.5
Ada Check your health	2.4	4	2	8.4
Apo Health Companion	3.2	1	4	8.2
Juva Stress and Migraine	3	3	2	8.0
Chronic Insights symptom diary	3.6	2	2	7.6
Lower Back Pain Exercises	3.3	2	2	7.3
Alogea	1.9	3	2	6.9
Purity Gout diet management	2.8	2	2	6.8
Symptom Tracker	2.6	3	1	6.6
mySymptoms Food Diary	3	2	1	6.0
#trackit: Track Health and Pain	2	1	2	5.0
Life notes—Symptom tracking	1.8	1	2	4.8

*Note*: Apps are presented highest‐lowest according to overall (mean) quality (MARS) rating. All tables have been organized from highest to lowest total composite rating.

Abbreviation: BCT, behaviour change technique; MARS, Mobile App Rating Scale (ref).

Taken together, these findings indicate that while most commercially available pain‐management apps include some evidence‐based features—particularly self‐monitoring and instructional content—only a minority offer a comprehensive, theoretically grounded approach. Notably, few apps appear to leverage the full potential of adaptive technologies to tailor interventions to individual needs in real time.

## DISCUSSION

This study presents evidence for a UK‐based systematic evaluation of commercially available pain management apps, examining app quality, pain‐relevant BCTs and adaptive features. Findings revealed substantial variability in overall app quality, with a small proportion of apps achieving good‐excellent quality (22%), and most apps (39%) achieving moderate quality. Only a small proportion achieved high subjective and app‐specific quality. Most apps included at least three pain‐relevant BCTs; however, certain BCT techniques such as social support and specific goal setting were rarely used. Adaptive mechanisms were inconsistently implemented; proximal outcomes were common across various apps, but tailoring and decision variables were infrequent.

Our findings regarding app quality align with prior research reporting that most commercially available pain‐management apps achieve moderate to excellent overall quality based on the MARS scale (Gamwell et al., 2021; Salazar et al., 2018). In these studies, functionality was often the highest‐rated domain. Zhou et al. (2024) reported a much lower mean overall quality score of 2.4 (poor quality) but similarly found functionality to be the strongest dimension in their review of back pain apps. The MARS overall score primarily reflects short‐term functional aspects of apps, including engagement, functionality, aesthetics and information quality, rather than sustained user engagement and behaviour change. This distinction is critical because sustained engagement has been acknowledged as a key determinant of positive pain self‐management outcomes (Bhatia et al., 2021). Our results revealed that apps generally scored lower on the MARS optional subscales (App Subjective and App Specific), which are closely linked to user satisfaction and the app's potential to promote target behaviours. While many apps meet functional quality standards, their capacity to engage users meaningfully over time and support effective pain self‐management may be limited. These results are consistent with earlier studies highlighting the oversimplification of many pain‐management apps and the lack of rigorous effectiveness testing (Lalloo et al., 2015; Portelli & Eldred, 2016; Rosser & Eccleston, 2011).

In terms of pain‐relevant BCTs, the most used techniques across the evaluated apps were: (i) self‐monitoring, (ii) instructions, and (iii) behaviour–health link. This indicates that app developers are integrating evidence‐based self‐management strategies known to positively influence pain‐related outcomes, which is consistent with recent findings. The importance of these specific BCTs is supported by research suggesting that self‐monitoring, instruction, and education are crucial in promoting engagement, improved self‐management and adherence to interventions, with ‘education’ here broadly reflecting BCTs such as instruction and behaviour–health link rather than a distinct taxonomy category (Chester et al., 2023). Additionally, modelling and education have been identified as important strategies in overcoming barriers to engagement in self‐management (Ziegler et al., 2025). Thus, the inclusion of pain‐relevant BCTs in commercially available pain‐management apps reflects alignment with behaviour change theory and incorporation of informed techniques. However, it is important to note that while research has found that using a higher number of BCTs is associated with an increase in the target behaviour, the type of BCT used, and how it is applied, may be more crucial for intervention design (Hankonen et al., 2015). Therefore, while our review reports the number of BCTs per app, this metric should not be interpreted as a proxy for effectiveness. The appropriateness, context and delivery of BCTs are likely more influential than quantity alone, and future research should examine these dimensions in detail.

Consistent with previous findings (Gamwell et al., 2021), our review revealed that two evidence‐based BCTs—specific goal setting and social support—were notably underutilized in pain‐management apps. Goal setting, which is essential for enhancing motivation, adherence and self‐efficacy (Bauer et al., 2016; Bovend'Eerdt et al., 2009), was present in only 17% (*N* = 4) of apps. Where implemented, features were often limited to generic prompts without personalized progress tracking or opportunities to review and adjust goals over time. Apps that score higher typically offered structured goal‐setting modules and a progress dashboard, whereas those scoring lower provided only static advice. To improve engagement and sustained behaviour change, future apps should incorporate dynamic goal‐setting frameworks, adaptive reminders and feedback loops.

Similarly, social support—a cornerstone of chronic pain self‐management programmes—was largely absent. Most apps lacked any form of peer interaction, community forums or links to external support networks, limiting opportunities for accountability and encouragement. This omission is concerning given the role of supportive accountability in adherence (Mohr et al., 2011) and the psychosocial challenges of chronic pain, including isolation and loneliness (Topcu, 2018). Evidence from mHealth interventions suggests that peer support can enhance motivation and self‐efficacy (Stenberg et al., 2022). Incorporating structured social features, such as moderated discussion boards, peer messaging or integration with caregiver support, could address these gaps and strengthen long‐term engagement. Findings from our related barriers and facilitators study reinforce this need: participants highlighted connection with like‐minded others and autonomy as key drivers of app engagement. Operationalizing goal‐setting and social support within app design is therefore critical for improving adherence and outcomes in chronic pain self‐management.

Overall, adaptive features were not commonly used in commercially evaluated apps: the highest number of features identified in a single app was 5 (Painalog) out of a possible 6, while 70% of apps included fewer than two. Evidence suggests that a variety of adaptable functions is most beneficial for digital health interventions (Hwang & Jiang, 2023; Kechagias & Papadopoulos, 2024), yet our review highlights a lack of diversity and an overall underutilization of such features in pain apps. Of the adaptive features assessed, proximal outcomes (immediate or short‐term goals) were the most consistently present, appearing in all apps, followed by intervention options (support content or strategies), which were found in 70% of the apps. These inclusions demonstrate some recognition of the value of actionable support and suggest that many apps incorporate at least a baseline level of adaptability.

In contrast, distal outcomes (longer‐term or ultimate goals) were found in fewer than half of apps and in some cases could only be inferred. This suggests that while apps often address short‐term needs, they less frequently articulate clinically meaningful long‐term objectives. Similarly, tailoring variables—mechanisms that allow interventions to adapt based on user data—were only sporadically implemented. Where present, these were usually basic forms of static personalization (e.g., baseline demographic information or notifications triggered after inactivity) rather than dynamic individualization based on real‐time monitoring of user states and contexts (Collins et al., 2004). Such limitations may partly reflect the practical and ethical challenges of collecting sensitive user data (Kjeldskov et al., 2004), but they also restrict the extent to which apps can deliver genuinely adaptive support. The rarity of tailoring variables was mirrored in the very limited use of decision points and decision variables, which typically require robust tailoring mechanisms as a foundation.

Just‐in‐Time Adaptive Interventions (JITAIs) have been proposed as a promising framework for delivering personalized digital health support by tailoring the type, timing and intensity of intervention content to an individual's real‐time state and context (Nahum‐Shani et al., 2015, 2016). Core elements of a JITAI include decision points (when the intervention can be delivered), tailoring variables (information about the user used to adapt the intervention), intervention options (types of support available), and proximal and distal outcomes (short‐ and long‐term goals). By using real‐time data to guide delivery, JITAIs aim to maximize relevance, responsiveness and effectiveness of digital interventions. JITAIs have already shown potential in areas such as physical activity, mental health and addiction interventions (Collins et al., 2024; Hardeman et al., 2019; Perski et al., 2022), where adaptability is critical to sustaining engagement and promoting meaningful behaviour change. Given the fluctuating and individualized nature of chronic pain, the application of JITAI principles could be particularly well suited to address the limitations we observed in UK published pain apps. For example, delivering personalized prompts, exercises or coping strategies at the moments they are most needed, JITAIs may enhance engagement, support real‐time self‐management and potentially facilitate sustained behaviour change over time. However, limitations exist: the requirement for continuous or frequent data collection may burden users, raise privacy concerns, or be affected by pain‐related cognitive or physical limitations. Additionally, the evidence for long‐term effectiveness of JITAIs in chronic pain remains limited, and their implementation requires careful consideration of which tailoring variables and decision points are clinically meaningful. Our findings suggest that while current apps incorporate some foundational adaptive elements, they fall far short of the dynamic personalization offered by JITAIs. JITAI‐informed approaches may be highly beneficial for advancing the design and effectiveness of digital pain self‐management interventions, particularly in supporting long‐term behaviour change through dynamic, responsive and personalized support. Future app development should prioritize accessibility, including availability in multiple languages and formats, to ensure broad usability across diverse populations. Ensuring apps are inclusive and easy to engage with, while retaining adaptive and personalized content, will be crucial to enhancing real‐world uptake and effectiveness in chronic pain populations.

### Strengths and limitations

This study aimed to replicate and extend the findings of previous investigations (Gamwell et al., 2021; Zhou et al., 2024), addressing a critical gap in the literature on digital pain self‐management interventions for non‐specific chronic pain conditions in the United Kingdom. We extended prior work by examining pain‐related BCTs in a UK context, moving beyond condition‐specific apps to include a broader range of pain‐management interventions, and by incorporating an assessment of adaptive features. In doing so, the study systematically evaluated core app features—quality, BCTs and adaptivity—that are known to influence behaviour change and adherence. Consequently, this research provides a more comprehensive overview of the current landscape of pain‐management apps than has previously been available. The use of established frameworks, including the MARS scale, the BCT taxonomy and adaptability principles, demonstrates a theory‐driven approach that enhances scientific rigour and enables meaningful comparisons with prior investigations. Additionally, a strength of this study is the rigour and replicability of the coding process. All apps were independently double coded by two clinical researchers with experience in behaviour change theory and intervention development. Coders interacted with each app for a minimum of 10 min to ensure a thorough evaluation of functionality and content. Discrepancies were resolved through open discussion, enhancing reliability and consistency. This structured yet flexible approach supports the credibility of the findings and offers a replicable framework for future evaluations of health apps. A further strength of this study is the inclusion of the optional MARS subscales—Subjective Quality and App‐Specific Quality, which, to our knowledge, were not assessed in previous evaluations (Gamwell et al., 2021; Zhou et al., 2024). Incorporating these subscales provided a more nuanced picture of app quality, capturing both coder‐perceived satisfaction and the perceived potential for behaviour change alongside the standard objective domains. This is particularly relevant in the context of health psychology and digital intervention uptake, as user satisfaction and perceived impact are key determinants of sustained engagement with digital health tools.

However, this study has several limitations. First, its cross‐sectional nature means that app features were assessed at a single point in time, rather than capturing longitudinal data on app updates, user retention or real‐world effectiveness. Consequently, this review could not evaluate whether app features or BCTs support sustained behaviour change or long‐term engagement, which is an important aspect of app functionality that MARS scoring alone cannot capture. Given the time‐limited interactions coders had with the apps, there is a possibility that certain BCTs or longer‐term dynamics were not fully captured during the evaluation period. Nonetheless, the duration of coder interaction followed recommended guidelines, that is, a minimum of 10 min per app (Stoyanov et al., 2015). BCTs were coded only for presence or absence following prior review for comparability, and as such, we did not assess the quality or fidelity of their implementation. This approach does not capture the specificity or appropriateness of the targeted behaviour, nor the context or delivery of technique. For example, instructions to rest versus instruction for graded activity would have very different implications for treatment efficacy. Due to our inability to determine how the inclusion of specific BCTs relates to app quality or effectiveness, future research should examine not only whether BCTs are present but also how they are framed and delivered (e.g. who, what, when, where and how) to determine whether they are theoretically sound and clinically meaningful. A further limitation relates to the search terms used. Because the review focused intentionally on apps marketed for general chronic pain self‐management, we did not include condition‐specific search terms (e.g., fibromyalgia, low back pain, arthritis, headache, cancer pain). While this was consistent with our aim and aligns with prior app evaluations, it is possible that high‐quality, condition‐specific pain apps were not captured. For example, it is plausible that condition‐specific apps, including those designed for cancer‐related pain, may differ in quality or feature sets, as such tools are often developed within clinical pathways, charitable organizations, or specialist services. However, because no condition‐specific apps appeared in our searches, we were unable to examine these potential differences within the present review. As such, the landscape described here reflects general pain apps only and may not represent condition‐targeted digital tools.

In addition, the set of pain‐relevant BCTs used in this study was based on a previously published framework (Gamwell et al., 2021), in which categories such as stress management and social support were necessarily broad. Although this allowed for replication and comparability, these broad categories limit the precision with which we can evaluate the nuance, quality or appropriateness of individual techniques within each domain. Consequently, some meaningful distinctions in BCT implementation may not have been detectable under this coding structure. Additionally, this evaluation was not exhaustive of all pain‐management apps and was limited to apps available in the UK app stores. Apps requiring a financial cost were excluded, broad search terms were employed, and only the first 25 apps from each store were included. This may be considered a key limitation that narrows the scope to free, publicly accessible tools. While this decision was justified by previous evidence highlighting cost as a significant barrier to engagement, it does mean that apps with a strong evidence base, such as *Curable © (£11.79/month, or £141.48 annually; discounted to £70.68 if paid upfront)*, were not included. *Curable*, for example, offers a multi‐modal, evidence‐informed approach incorporating psychoeducation, stress management, and other validated BCTs, and has demonstrated effectiveness in academic studies (Devan et al., 2019; Thomson et al., 2024). As such, this review captures only the landscape of free apps and may not reflect the full range of high‐quality digital interventions available to pain patients who can afford, and are willing to, pay for app access. However, restricting such interventions to those able to afford subscriptions risks widening health inequalities, particularly as people with chronic pain are disproportionately represented among socioeconomically disadvantaged groups (Dahlhamer et al., 2023; Grol‐Prokopczyk, 2017). However, this search strategy was consistent with prior published evaluations and reflects the typical app‐discovery process used by consumers (Chavez et al., 2017; Gamwell et al., 2021; Vaghefi & Tulu, 2019).

## CONCLUSIONS

This study provided a systematic evaluation of commercially available and free to download pain‐management apps in the United Kingdom and assessed app quality, presence of BCTs and adaptive features. The findings suggest substantial variability in app quality and integration of evidence‐based, pain‐relevant BCTs and adaptive mechanisms. Although apps demonstrated moderate to high functional quality overall, essential BCTs such as social support and prompt‐specific goal setting were underutilized, and adaptive features were limited in scope. These factors may restrict apps' ability to meaningfully engage users and promote adherence, both of which are critical for effective pain self‐management and relevant within the context of chronic pain. Future research and app development should prioritize the integration of theory‐informed BCTs and adaptive features, including dynamic tailoring and decision points, to enhance user engagement and support sustained behaviour change in chronic pain management.

## AUTHOR CONTRIBUTIONS

**Rebecca P. Harding:** Conceptualization; methodology; investigation; formal analysis; project administration; writing – original draft; data curation; visualization. **Jenna L. Gillett:** Conceptualization; methodology; data curation; formal analysis; validation; writing – review and editing. **Michael Passaportis:** Conceptualization; methodology; validation; writing – review and editing; supervision. **Eleanor Miles:** Conceptualization; methodology; validation; writing – review and editing; supervision. **Faith Matcham:** Conceptualization; software; validation; supervision; visualization; project administration; funding acquisition; resources; writing – review and editing.

## CONFLICT OF INTEREST STATEMENT

No conflicts of interest.

## REGISTRATION

The protocol was prospectively registered on the Open Science Framework (OSF) in February 2024: *A systematic review: Evaluation of pain‐management applications (apps) for chronic pain in the UK* (doi: https://doi.org/10.17605/OSF.IO/2WUJ8).

## Supporting information


Data S1.

## Data Availability

All data were obtained from publicly accessible app store listings. No new data were generated.
