# Supplementary material for: Systematic evaluation of commercially available pain‐management mHealth apps for chronic pain in the United Kingdom
Source: Br J Health Psychol. 2026 Jan 26;31(1):e70053. doi: 10.1111/bjhp.70053 (PMC12835579; doi:10.1111/bjhp.70053)
Supplement: Supplementary file 1 — Data S1. [file BJHP-31-0-s001.docx]

*Supplementary material 1. Changes to registered protocol*

The preregistered protocol specified broader search terms (e.g., “chronic pain,” “pain management,” “chronic pain patients,” “CPPs,” “pain,” “reduce pain”) in combination with app-related terms (e.g., “application,” “app,” “mHealth,” “just-in-time adaptive intervention”). During piloting, this approach retrieved a large volume of irrelevant results. To improve feasibility and specificity, we refined the search strategy to focus on pain-related terms only (“chronic pain,” “pain management,” “chronic pain patients,” “CPPs,” “reduce pain”), entered directly into the Apple and Google Play store search engines. This refinement produced a more manageable and relevant set of apps, consistent with approaches used in previous app evaluations (e.g., Gamwell et al., 2021). In addition, the preregistered protocol stated that apps targeting a specific pain condition (e.g., arthritis, lower back pain) would be excluded. This criterion was revised during the review to include condition-specific apps where pain management was a core focus. This change was made to provide a more comprehensive overview of commercially available pain-management apps and to reflect the reality of how patients may search for and use apps.
